# Physician Attitudes Towards Pharmacist-Prescribed HIV Post-Exposure Prophylaxis (PEP): A Survey of a State Medical Association

**DOI:** 10.1007/s10900-024-01421-x

**Published:** 2024-12-04

**Authors:** Kaylee Scarnati, Katherine L. Esser, Julianna M. Sim, Varun Vaidya, Eric Sahloff, Joan Duggan

**Affiliations:** 1 University of Toledo College of Medicine and Life Sciences, Toledo, OH USA; 2 University of Toledo College of Pharmacy and Pharmaceutical Sciences, Toledo, OH USA; 3 Department of Infectious Disease, University of Toledo Medical Center, University of Toledo College of Medicine and Life Sciences, Toledo, OH USA

**Keywords:** Post-exposure prophylaxis, HIV, Medical trainees, Pharmacist-prescribed

## Abstract

Timely initiation of antiretroviral therapy (ART) for non-occupational post-exposure prophylaxis (nPEP) is crucial in preventing HIV infection and advancing efforts to end the HIV epidemic (EHE). nPEP must be administered within 72 h of high-risk exposure, ideally within 24 h. Pharmacies may play a role in increasing access to nPEP and facilitating referrals for additional care, such as PrEP. Recent legislation permits pharmacist-prescribed nPEP (PDP), though provider attitudes toward this change have not been studied. A survey querying physicians and medical trainees (students and residents) was conducted in 2024 during an annual state medical association conference. The survey included 24 questions on nPEP knowledge and attitudes towards pharmacist-prescribed nPEP (PDP). The survey was administered in person using electronic tablets, with voluntary participation incentivized by a monetary reward. Statistical analysis was conducted using SAS (9.4 version) software, with differences in responses between physicians and trainees evaluated. P values < 0.05 were considered statistically significant. Of 89 respondents (56% of attendees), 61 were physicians and 28 were trainees. Notably, 28% of licensed providers indicated that pharmacist-directed prescribing (PDP) should not be allowed in the State of Ohio, while no trainees expressed this same opposition (p = 0.001). Trainees were more likely to view pharmacist prescribing as safe and appropriate ((p = 0.0135, p = 0.013). Urban-based providers were more supportive of pharmacist prescribing than their rural counterparts (p = 0.0195). Trainees showed little opposition to PDP, whereas physicians expressed concerns about safety and appropriateness. Addressing these concerns is essential for integrating pharmacists into the EHE strategy.

**Supplementary Information:**

The online version contains supplementary material available at 10.1007/s10900-024-01421-x.

## Introduction

The HIV epidemic has been one of the most significant global health challenges of the last 40 years [1]. It has killed more than 700,000 people in the United States alone, and has disproportionately affected minority communities [2]. The Ending HIV Epidemic (EHE) campaign launched in 2019 by the US Department of Health and Human Services, aims to reduce the incidence of annual HIV infections by 90% by 2030 [3, 4]. The US National HIV/AIDS Strategy (NHAS) set specific goals in 2021 for the EHE initiative: to prevent new infections, improve HIV-related outcomes, and reduce HIV-related health disparities [5].

A key strategy to stop HIV transmission is the use of HIV antiretroviral therapy (ART) for post exposure prophylaxis (PEP). PEP is a 28-day course antiretroviral therapy that must be initiated with 72 h (ideally 24 h) after a potential or known exposure to HIV to prevent subsequent infection. Prompt access to PEP is crucial to maximize its preventative potential [6]. Providing PEP after a known/potential exposure to HIV at the workplace (ex. Healthcare workers) is called occupational PEP. Should possible occupational exposure occur, institutional policies and procedures are generally in place for timely testing and provision of occupational PEP based on current practice guidelines. Non-occupational PEP (nPEP) is provided to individuals with known or potential exposure to HIV through sexual contact or injection drug use. Responsibility is on the individual to seek out nPEP in these situations, which can often lead to prohibitive delays in seeking and receiving appropriate care. Despite its importance, urgent access to nPEP poses significant challenges such as lack of public and provider awareness or comfort with nPEP regimens in acute care settings [7]. Additionally, a 2020 survey found that 13% of providers in non-acute care settings were unaware of nPEP, and 44% knew of nPEP, but had never prescribed it [8]. This is a concerning finding given that non-acute care centers are often the primary points of contact for individuals seeking nPEP. The disparity between PEP efficacy and utilization suggests that identifying strategies for improving access is essential in order to effectively use this intervention to help end the HIV epidemic.

In 2017, New York City pioneered the expansion of nPEP access through pharmacies, allowing pharmacists to prescribe a seven-day supply of nPEP without a prescription for consumers at high risk for HIV infection [9]. This initiative aimed to leverage the broad reach and accessibility of community pharmacies to improve timely access to nPEP, especially for those facing barriers to traditional healthcare [10]. Since then, several states have expanded the scope of pharmacy practice to include nPEP, but over 75% of prescribing laws remain unchanged [9]. Pharmacist-driven PEP (PDP) enhances immediate access to ART and can also facilitate referrals for ongoing preventive care. There is a paucity of data regarding physician knowledge and attitudes towards PDP [7, 8, 11]. Understanding these attitudes is crucial, as physician support and collaboration are vital for the successful implementation of PDP programs.

This study seeks to address this gap by examining the perspectives of physicians and medical trainees on the appropriateness and safety of PDP. Conducted through a survey of members of the Ohio State Medical Association (OSMA), this research aims to elucidate the level of support and concerns about this emerging model of care. The OSMA, a prominent state medical society, comprises physicians and medical trainees who are deeply engaged in medical advocacy and policy-making. Their active involvement in shaping healthcare policies makes them an ideal cohort for assessing attitudes towards innovative healthcare delivery models like PDP among current and future medical practitioners. Surveying this particular group provides valuable insights from individuals who are not only frontline healthcare providers but also influential voices in medical policy and advocacy.

## Methods

### Participants

A convenience sample of licensed physicians (active, retired, and fellows) and medical trainees (medical students, this group was originally intended to also include Residents, however no Residents participated in this study) who are members of a state medical society (Ohio State Medical Association—OSMA), which has a membership of 9000, were surveyed at an annual meeting (April 2024 in Columbus, Ohio). While medical residents were originally intended to be included in the trainee category, none participated, so the term 'medical trainees' throughout this manuscript refers only to medical students. Participation was voluntary and anonymous, and a small monetary gift card was offered to participants after survey completion. Participation required informed consent. The study was approved by the Institutional Review Board (IRB) of the University of Toledo, Toledo, OH (IRB #301846UT). A complete copy of the survey is included in online Appendix 1.

### Procedures

Study personnel offered the self-administered survey to potential participants during meeting hours. The survey was conducted using the Qualtrics platform which also stored survey data. The survey was provided electronically via QR code or tablet to interested participants. The first page of the survey consisted of informed consent information; initiating the survey was considered consent to participate. Only one survey was accepted per participant, and estimated time to completion was between 5 and 10 min.

### Survey Questions

The branching survey consisted of 24 to 27 questions which varied depending on practice status (medical students, residents, fellows, attendings, retired physicians). The survey is provided as online Appendix 1. Respondents who identified as trainees were not asked questions about active medical practice. Questions fell into the following domains:

- Demographics: Age, gender, sexual orientation, race, zip code of practice or training setting, and status of practice (medical student, resident, fellow, attending, retired). Fellows and physicians had additional questions regarding specialty and subspecialty, primary practice setting (inpatient, outpatient, urgent care, or emergency room), and retired physicians were asked about time since they last practiced.
- Non-occupational PEP (nPEP) knowledge: A paragraph providing foundational information on nPEP was included in the survey for two primary purposes. First, it ensured that all participants had a baseline level of knowledge necessary to accurately respond to certain survey questions, as a basic understanding of nPEP was required for informed answers. Second, the paragraph served as a tool to assess participants' self-assessment of their pre-existing knowledge, as they were asked to indicate whether they were already familiar with the provided information. This was followed by questions evaluating participants’ knowledge of nPEP, their involvement in the care of individuals living with HIV, and their experience with nPEP delivery over the past year.
- Pharmacist-prescribed medications: A paragraph of basic information about state and federal law regarding pharmacist-prescribed emergency medications [emergency naloxone, emergency contraception, immunizations, nirmatrelvir/ritonavir (Paxlovid®) for COVID-19 under FDA guidance] was provided. Participants were asked to use a 5-point Likert Scale to rate their perceptions of the importance of timely nPEP and occupational PEP, the safety and appropriateness of pharmacist prescribing of emergency medications and nPEP, the impact of pharmacist prescribing of nPEP on access and appropriate use of nPEP, and the impact on diagnosis of sexually transmitted infections and follow up medical care. Finally, they were asked how, if at all, pharmacists should be allowed to prescribe nPEP.

### Statistical Analysis

Statistical analysis was performed using SAS (9.4 version) software. Descriptive statistics summarized demographic characteristics and preferences. Chi-square tests were performed to determine if there were significant associations between various Likert scale variables, restructured into three categories (Positive, Neutral, and Negative), and the current practice categories. For the purposes of the analysis, medical students and residents were grouped together (trainee group) and fellows, attendings, and retired physicians were grouped together (licensed group).

## Results

### Demographics

Respondent demographics are reported in Table 1. Surveys were completed by 89 out of 160 attendees (56%), including 28 medical students, 0 residents, 49 attendings, 1 fellow, and 11 retired physicians. The licensed provider group was approximately 65% white male and almost entirely heterosexual (98%), with a mean age of 54.2 years (SD: 24.7). In contrast, the trainee group was more likely to be female (57%), white (67%), and reported sexual orientation as straight (64%), gay/lesbian (18%), and bisexual (11%). Sexual orientation and gender were significantly different for licensed providers compared to trainees. Licensed providers practiced primarily in the urban setting (79%), while students were fairly split between urban (57%) and rural (43%) training locations. Race and practice/training location did not differ significantly between the licensed provider group and trainees. Among the 49 licensed providers, active practice settings were reported as emergency room (6/49), inpatient (10/49), and outpatient (33/49). Family medicine (9/50), internal medicine (8/50), obstetrics/gynecology (8/50), and emergency medicine (5/10) represented the most commonly reported specialties.

**Table 1:** Characteristics of Survey Respondents (n = 89)

Demographic category	Licensed providers	Trainees	Total	P-Value
Age: Mean (SD)	54.2 (24.7)	25.7 (1.98)	78	–
Gender				0.0159*
Female	20 (33.3%)	16 (57%)	36	–
Male	39 (65%)	10 (35.7%)	49	–
Non-binary/third gender	0	2(7.1%)	2	–
Prefer Not to say	1(1.7%)	0	1	–
Sexual orientation				0.0001*
Bisexual	1(1.7%)	3(10.7%)	4	–
Lesbian or Gay	0	5(17.9%)	5	–
Straight	57(98.3%)	18(64.3%)	75	–
Uses a different term	0	2(7.1%)	2	–
Race/Ethnicity				0.591
American Indian/Alaskan Native, non-hispanic	1(1.7%)	0	1	–
Asian, non-hispanic	11(19.0%)	6(21.4%)	17	–
Black or African American	5(8.6%)	2(7.1%)	7	–
Hispanic	0	1(3.6%)	1	–
White, non-hispanic	37 (63.8%)	19(67.9%)	56	–
Multiracial, nonhispanic?	2(3.4%)	0	2	–
Other race, non-hispanic	2(3.4%)	0	2	–
Practice region of state				0.151
Central	14	2	16	–
Northeast	21	9	30	–
Northwest	14	6	20	–
Southeast	1	3	4	–
Southwest	10	8	18	–
Not Reported	1	0	1	–
Knowledge of PEP				0.1395
Aware of all the provided information	19(31.1%)	3(10.7%)	22	–
Aware of most of the information	14(22.9%)	11(39.3%)	25	–
Aware of some of the information	24(39.3%)	11(39.3%)	35	–
Not aware of this information	4(6.6%)	3(10.7%)	7	–
Knowledge self-ranking				0.438
Expert	2(3.3%)	0	2	–
Advanced	7(11.5%)	4(14.3%)	11	–
Intermediate	30 (49.2%)	9 (32.1%)	39	–
Novice	17 (27.9%)	12 (42.9%)	29	–
No Experience	5 (8.2%)	3 (10.7%)	8	–
People living with HIV cared for in last year				
1–10	31 (51.7%)	15 (53.6%)	46	
11–25	6 (10%)	0	6	
26–50	4 (6.7%)	0	4	
> 50	3 (5%)	0	3	
None	16 (26.7%)	13 (46.4%)	29	
Patients eligible for nPEP cared for in last year				
1–10	22 (44%)	5 (17.9%)	27	
11–25	4 (8%)	0	4	
26–50	1 (2%)	0	1	
None	23 (46%)	23 (82.1%)	46	

### Knowledge and Experience

Respondent knowledge and experience are detailed in Table 1. Regarding self-assessment of nPEP knowledge, both groups were primarily intermediate or novice (providers 77%, trainees 75%), with approximately 8–10% in each reporting no experience. Half of the licensed provider group reported caring for 1–10 people living with HIV (PWH) in the last year and approximately one-quarter reported no experience with PWH. Similarly, just over 50% of trainees reported participating in the care of PWH, with the remainder having no experience.

### Preferences/Attitudes

Survey responses regarding attitudes towards nPEP, occupational PEP, and pharmacist prescribing are summarized in Tables 2–5. Table 3 specifically compares medical trainees to licensed providers in their attitudes toward nPEP. Over 90% of both trainees and licensed physicians agreed that providing timely nPEP and occupational PEP were important, while significantly more trainees felt that allowing pharmacists to prescribe nPEP would improve access (85.7% vs 57.6%, p = 0.0339). Trainees were significantly more likely to respond that pharmacists prescribing urgent medications and nPEP were both safe and appropriate.

**Table 2:** Comparison of Preferences and Perceived Impact of Pharmacist-Driven nPEP for HIV (n = 89*)

	Medical trainees (MT)	Licensed providers (LP)	P value
What type of system for nPEP would work well in the state?			0.0017*
Pharmacists allowed to provide 28-day script for PEP using State Board of Pharmacy-approved protocol	7/61 (11.5%)	11/28 (39.3%)	–
Pharmacists allowed to provide 7-day script for PEP using State Board of Pharmacy-approved protocol with mandatory requirement for referral/linkage to medical care	32/61 (52.5%)	15/28 (53.6%)	–
Pharmacist should not be allowed to prescribe PEP in state	17/61 (27.9%)	0/28 (0%)	–
Other	5/61 (8.2%)	2/28 (7.1%)	–
What do you think would be the impact on appropriate use of PEP if pharmacists are allowed to prescribe nPEP			0.0659
Decrease inappropriate use	5/28 (17.9%)	5/61 (8.2%)	–
Increase inappropriate use	8/28 (28.6%)	33/61 (54.1%)	–
No impact on inappropriate use	15/28 (53.6%)	23/61 (37.7%)	–
What do you think will be the impact on the diagnosis of HIV and/or sexually transmitted infections (STIs) if pharmacists prescribe nPEP?			0.8086
Decrease diagnosis of HIV and/or STIs	14/28 (50%)	28/60 (46.7%)	–
Increase diagnosis of HIV and/or STIs	7/28 (25%)	13/60 (21.7%)	–
No impact on diagnosis of HIV and/or STIs	7/28 (25%)	19/60 (31/7%)	–
What do you think will be the impact on medical follow up for care for people at risk of HIV infection if pharmacists prescribe nPEP?			0.0019*
Decrease follow up	9/28 (32.1%)	42/61 (68.9%)	–
Increase follow up	13/28 (46.4%)	9/61 (14.8%)	–
No impact on follow up	6/28 (21.4%)	10/61 (16.4%)	–

^*^While there were a total of 89 respondents, not every question received responses from all of them. This is due to skipped questions or the branching design of the survey, which made some questions irrelevant for certain respondents. The actual number of respondents for each question is indicated in the denominator of each fraction

**Table 3:** Medical Trainees (MT) and licensed provider (LP) attitudes toward PEP use and pharmacist prescribing of PEP (n = 89*)

	Level	Disagree	Neutral	Agree	P value
Important to provide timely PEP	MT	1/28 (3.6%)	1/28 (3.6%)	26/28 (92.9%)	0.278
	LP	0/61 (0%)	1/61 (1.6%)	60/61 (98.4%)	
Important to provide occupational PEP	MT	0/27 (0%)	2/27 (7.4%)	25/27 (92.6%)	0.391
	LP	0/61 (0%)	2/61 (3.3%)	59/61(96.7%)	
Allowing pharmacists to prescribe PEP will improve access	MT	2/28 (7.1%)	2/28 (7.1%)	24/28 (85.7%)	0.034*
	LP	14/59(23.7%)	11/59 (18.6%)	34/59 (57.6%)	
Allowing pharmacists to prescribe medications that require urgent administration such as Narcan or Paxlovid to patients who have not seen a physician or physician extender is safe	MT	1/28 (3.6%)	5/28 (17.9%)	22/28 (78.6%)	0.006*
	LP	19/61(31.1%)	14/61 (23.0%)	28/61(45.9%)	
Allowing pharmacists to prescribe medications that require urgent administration such as Narcan or Paxlovid to patients who have not seen a physician or physician extender is appropriate	MT	2/28 (7.1%)	5/28 (17.9%)	21/28 (75%)	0.037*
	LP	19/61(31.1%)	11/61 (18.0%)	31/61(49.2%)	
Allowing pharmacists to prescribe PEP to patients who have not seen a physician or physician extender is safe	MT	3/28 (10.7%)	3/28 (10.7%)	22/28 (78.6%)	0.014*
	LP	23/60(38.3%)	9/60 (15.0%)	28/60 (46.7%)	
Allowing pharmacists to prescribe PEP to patients who have not seen a physician or physician extender is appropriate	MT	5/28 (17.9%)	3/28 (10.7%)	20/28 (71.4%)	0.014*
	LP	25/58	11/58 (19.0%)	22/58 (37.9%)	

^*^While there were a total of 89 respondents, not every question received responses from all of them. This is due to skipped questions or the branching design of the survey, which made some questions irrelevant for certain respondents. The actual number of respondents for each question is indicated in the denominator of each fraction

Using the question “What type of system for nPEP would work well in Ohio?”, preferences of trainees and providers are summarized in Table 2. Seventeen of 61 of licensed providers indicated that pharmacists should not be allowed to prescribe in Ohio, compared to 0% of trainees submitting this same response. Allowing pharmacists to provide a 7-day prescription of nPEP (per state board of pharmacy approved protocol) with mandatory linkage to medical care was selected by 32 out of 61 (52%) of licensed physicians and 15 out of 28 (54%) of trainees. Fewer respondents from both groups preferred allowing pharmacists to provide a 28-day prescription for nPEP (7 out of 61 (11%) of licensed providers and 11 out of 28 (39%) of trainees (p = 0.0017). These differences of opinion between medical trainees and licensed providers regarding the extent of pharmacists' authority to prescribe PEP were statistically significant (p = 0.0017). Additionally, although not statistically significant, almost twice as many licensed providers tended to feel that pharmacist prescribing of nPEP would increase inappropriate use compared to trainees [33/61 (54.1%) vs 8/28 (28.6%), p = 0.0659] and nearly half of both trainees and licensed providers felt that pharmacist prescribing of nPEP would decrease the diagnosis of HIV [14/28 (50%) vs 28/60 (46%), p = 0.8086]. Over twice as many licensed providers [42/61 (68.9%)] compared to trainees [9/28 (32.1%)] felt that pharmacist prescribing of nPEP would decrease medical follow up for people at risk for acquiring HIV (p = 0.0019).

Urban based licensed providers were significantly more likely to agree that pharmacist prescribing of urgent medications and nPEP was safe (Urgent meds: 62.5% vs 46.9%, p = 0.0224; nPEP: 64.3% vs 41.9%, p 0.0195), compared to their rural counterparts. (Table 4) However, there was no significant difference in perceived appropriateness of pharmacists prescribing these medications (urgent medications: 64.3% vs 41.9%, p = 0.0756; nPEP: 54.5% vs 40.0%, p 0.4195). Table 5 compares responses from individuals who had cared for at least one patient requiring nPEP versus those who had not. A similar number of respondents from both groups (~ 50–60%) felt that pharmacists prescribing urgent medications or PEP was safe and appropriate which was not significantly different between groups.

**Table 4:** Respondents attitudes toward PEP use and pharmacist prescribing of PEP based on practice/training in rural versus urban location (n = 89*)

	Location	Disagree	Neutral	Agree	P value
Important to providing timely PEP	Rural	0/32 (0%)	0/32 (0%)	32/32 (100%)	0.412
	Urban	1/56 (1.8%)	2/56 (3.6%)	53/56 (94.6%)	
Important to provide occupational PEP	Rural	0/32 (0%)	1/32 (3.1%)	31/32 (96.9%)	0.612
	Urban	0/55 (0%)	3/55 (5.5%)	52/55 (94.5%)	
Allowing pharmacists to prescribe PEP will improve access	Rural	2/26 (7.7%)	5/26 (19.2%)	19/26 (73.1%)	0.815
	Urban	6/53 (11.3%)	8/53 (15.1%)	39/53 (73.6%)	
Allowing pharmacists to prescribe medications that require urgent administration such as Narcan or Paxlovid to patients who have not seen a physician or physician extender is safe	Rural	12/32 (37.5%)	5/32 (15.6%)	15/32 (46.9%)	0.022*
	Urban	7/56 (12.5%)	14/56 (25%)	35/56 (62.5%)	
Allowing pharmacists to prescribe medications that require urgent administration such as Narcan or Paxlovid to patients who have not seen a physician or physician extender is appropriate	Rural	12/32 (37.5%)	5/32 (15.6%)	15/32 (46.9%)	0.076*
	Urban	9/56 (16.1%)	11/56(19.6%)	36/56 (64.3%)	
Allowing pharmacists to prescribe PEP to patients who have not seen a physician or physician extender is safe	Rural	15/31(48.4%)	3/31 (9.7%)	13/31 (41.9%)	0.020*
	Urban	11/56 (19.6%)	9/56 (16.1%)	36/56 (64.3%)	
Allowing pharmacists to prescribe PEP to patients who have not seen a physician or physician extender is appropriate	Rural	13/30 (43.3%)	5/30 (16.7%)	12/30 (40.0%)	0.420
	Urban	17/55 (30.9%)	8/55 (14.5%)	30/55 (54.5%)	

^*^While there were a total of 89 respondents, not every question received responses from all of them. This is due to skipped questions or the branching design of the survey, which made some questions irrelevant for certain respondents. The actual number of respondents for each question is indicated in the denominator of each fraction

**Table 5:** Comparison of respondents who have never prescribed PEP vs those who have prescribed PEP more than once (n = 89*)

	Level	Disagree	Neutral	Agree	P value
Important to providing timely PEP	None	1/51 (2.0%)	2/51 (3.9%)	48/51 (94.1%)	0.315
	≥ 1	0/38 (0%)	0/38 (0%)	38/38 (100%)	
Important to provide occupational PEP	None	0/50 (0%)	3/50 (6%)	47/50 (94%)	0.452
	≥ 1	0/38 (0%)	1/38 (2.6%)	37/38 (97.4%)	
Allowing pharmacists to prescribe PEP will improve access	None	6/50 (12%)	9/50 (18%)	35/50 (70%)	0.170
	≥ 1	10/37 (27.0%)	4/37 (10.8%)	23/37 (62.2%)	
Allowing pharmacists to prescribe medications that require urgent administration such as Narcan or Paxlovid to patients who have not seen a physician or physician extender is safe	None	9/51 (17.6%)	11/51 (21.6%)	31/51 (60.8%)	0.429
	≥ 1	11/38 (28.9%)	8/38 (21.1%)	19/38 (50%)	
Allowing pharmacists to prescribe medications that require urgent administration such as Narcan or Paxlovid to patients who have not seen a physician or physician extender is appropriate	None	9/51 (17.6%)	10/51(19.6%)	32/51 (62.7%)	0.309
	≥ 1	12/38 (31.6%)	6/38 (15.8%)	20/38 (52.6%)	
Allowing pharmacists to prescribe PEP to patients who have not seen a physician or physician extender is safe	None	15/51 (29.4%)	7/51 (13.7%)	29/51 (56.9%)	0.999
	≥ 1	11/37 (29.7%)	5/37 (13.5%)	21/37 (56.8%)	
Allowing pharmacists to prescribe PEP to patients who have not seen a physician or physician extender is appropriate	None	16/49 (32.7%)	7/49 (14.3%)	26/49 (53.1%)	0.652
	≥ 1	14/37 (37.8%)	7/37 (18.9%)	16/37 (43.2%)	

^*^While there were a total of 89 respondents, not every question received responses from all of them. This is due to skipped questions or the branching design of the survey, which made some questions irrelevant for certain respondents. The actual number of respondents for each question is indicated in the denominator of each fraction

### Consistency in Responses

An analysis of responses regarding the appropriateness and safety of pharmacist-provided urgent medications and nPEP. The result showed a high level of consistency and significance (p < 0.001). For example, those who viewed pharmacist-provided urgent medications, such as naloxone, as safe also viewed pharmacist-provided nPEP as safe.

## Discussion

The most important findings from this survey reveal that significant differences exist between licensed providers and medical trainees in their attitudes towards pharmacist-prescribed HIV nPEP. Trainees were more likely to support pharmacist-driven nPEP, viewing it as both safe and appropriate. A significant proportion of trainees agreed that pharmacist-prescribed nPEP was safe prior to seeing a physician (78.6%), whereas only 45.9% of providers concurred (p = 0.014). Similarly, 71.4% of trainees felt pharmacist-prescribed nPEP prior to seeing a physician was appropriate, while only 37.9% of providers shared this view (p = 0.014).

We categorized respondents into two groups: "licensed providers" (which includes licensed physicians, fellows, and retired physicians) and "medical trainees" (which consisted of medical students). This division was intended to reflect varying levels of experience and perspectives within the medical community, offering insights into how attitudes towards pharmacist-prescribed PEP differ based on professional status and generational shifts in thinking. Providers, with their extensive clinical experience and adherence to more traditional medical practices, were compared to trainees, who are in the formative stages of their education and may be more open to innovative approaches in healthcare, including interprofessional collaboration. This generational shift in attitudes may be further supported by trainees' exposure to formal interprofessional training, which may make them more receptive to expanded pharmacist roles [12, 13].

Statistical analyses also stratified respondents by practice location to identify geographical differences in attitudes towards pharmacist-driven PEP (PDP). Urban-based providers were significantly more likely to view PDP as safe compared to their rural counterparts (62.5% vs. 46.9%, p = 0.022). This may be influenced by the greater availability and integration of pharmacists into healthcare systems in urban areas, where collaborative care models are common [9]. Urban providers may also have more experience working alongside pharmacists, fostering greater trust in their ability to manage urgent medications like nPEP. In contrast, rural providers, who may be accustomed to traditional, physician-led care models, may have limited exposure to the expanded roles of pharmacists, contributing to their hesitancy. Interestingly, while there was a significant difference in perceptions of safety, the perceived appropriateness of pharmacist prescribing was low in both groups and did not differ significantly between urban and rural providers (54.5% vs. 40.0%, p = 0.420). This suggests that although urban providers may recognize the safety of PDP, concerns about its broader implementation remain, possibly due to challenges in healthcare infrastructure and continuity of care in rural settings.

In addition to geographical differences, this study also examined the impact of previous experience with nPEP on attitudes towards PDP. Respondents were divided into those who had never prescribed nPEP and those who had prescribed nPEP more than once. Surprisingly, there were no significant differences in attitudes towards the safety and appropriateness of pharmacist-driven PEP between these two groups. This suggests that attitudes may be driven more by skepticism about expanding pharmacist roles than by clinical experience with nPEP itself.

The results of this research underscores the need for targeted educational initiatives into nPEP and prescription by pharmacists. Approximately 77% of providers and 75% of trainees self-reported being at an intermediate or novice level of understanding of PEP, with 8–10% in each group reporting no experience with PEP at all. This finding builds on previous assessments of provider knowledge of nPEP which found similarly low awareness of the intervention [14]. This knowledge gap points to a significant area for improvement in medical education and continuing professional development. While not all providers regularly treat HIV positive patients, the wide range of individuals at risk necessitates that all providers have an awareness and understanding of nPEP as an intervention. By doing so, healthcare professionals will be better equipped to identify at-risk patients and connect them with appropriate resources. Enhancing familiarity with nPEP’s efficacy, and safety profile is essential to build confidence in the expanded role of pharmacists in HIV prevention. Moreover, targeted educational initiatives are important for improving access to nPEP and addressing the concerns of providers regarding PDP programs, fostering a more cohesive, interdisciplinary approach to HIV prevention and care.

To gauge consistency in beliefs, respondents were also asked about their attitudes towards pharmacist prescription of other urgent medications, such as naloxone and Paxlovid. The results indicated that providers who were skeptical about pharmacist prescription of nPEP were just as likely to hold reservations across other areas of prescriptive authority for pharmacists. This finding suggests that integrating pharmacists into the HIV care continuum should be part of a broader strategy to promote interdisciplinary collaboration and address concerns about pharmacist prescribing across the board.

The implications of these findings are significant as the U.S. strives to end the HIV epidemic by 2030. Expanding the role of pharmacists could improve timely access to nPEP, especially in underserved areas, but addressing the concerns of licensed providers is critical for the successful implementation of pharmacist-driven PEP programs. Educational initiatives aimed at both trainees and licensed providers could help bridge the knowledge gap identified in this study, where both groups reported intermediate or novice levels of understanding of nPEP. Increasing familiarity with the efficacy and safety of PEP and fostering interdisciplinary collaboration will be essential to overcoming barriers and ensuring that all patients have access to comprehensive HIV prevention services. As the U.S. moves towards the 2030 goal of ending the HIV epidemic, pharmacist-driven PEP will be looked to as the potential key to expanding access and improving outcomes in HIV care.

This study had several limitations. The sample size was relatively small, and the survey was conducted at a single state medical association conference, which could introduce selection bias and limit the generalizability of the findings to the broader U.S. medical community. Further, the survey relied on self-reported data, which can be subject to recall bias or social desirability bias, particularly when assessing knowledge and attitudes. Exploration of these limitations are all areas of possible future study.

## Conclusion

Our findings reveal significant differences in attitudes towards pharmacist-driven PEP between licensed providers and trainees, with trainees generally showing more support for pharmacist involvement. The study highlights the need for continued dialogue and collaboration between pharmacists and licensed providers to integrate pharmacists effectively into EHE and the HIV care continuum. As the role of pharmacists evolves, addressing these differences in perception and experience will be crucial for enhancing access to and delivery of HIV prevention and treatment services.

## Supplementary Information

Below is the link to the electronic supplementary material.

Supplementary file1 (DOCX 26 kb)

## Data Availability

Data collection was performed by Kaylee Scarnati, Julianna Sim and Katherine Esser. The first draft of the manuscript was written by Kaylee Scarnati, Julianna Sim and Katherine Esser, and all authors participated in revisions of the manuscript. Statistical analysis was performed by Varun Vaidya. All authors read and approved the final manuscript.
