# Supplementary material for: Physician Attitudes Towards Pharmacist-Prescribed HIV Post-Exposure Prophylaxis (PEP): A Survey of a State Medical Association
Source: J Community Health. 2024 Dec 4;50(2):335–43. doi: 10.1007/s10900-024-01421-x (PMC11937200; doi:10.1007/s10900-024-01421-x)
Supplement: Supplementary file 1 — Supplementary file1 (DOCX 26 kb) [file 10900_2024_1421_MOESM1_ESM.docx]

Appendix 1. Copy of Survey.

Start of Block: Disclaimer

**Q85**
 **Department of Medicine** 3000 Arlington Avenue, MS
 Toledo, Ohio 43614


 **ADULT RESEARCH SUBJECT - INFORMED CONSENT FORM** ***Attitudes of medical students, residents, and physicians regarding pharmacy-driven post-exposure prophylaxis for HIV in Ohio*** **Principal Investigator** *Joan Duggan, MD, AAHIVE, 419-383-4328*
 ***Other Investigators*** *Eric Sahloff, PharmD, AAHIVP, 419-383-1962*  *Katherine Esser, MD Candidate 2025, 440-223-3646*  *Kaylee Scarnati, MD Candidate 2025, 330-984-8692*  *Julianna Sim, MD Candidate 2025, 419-345-9242*
 **Purpose:** You are invited to participate in the research project entitled *“Attitudes of medical students, residents, and physicians in regarding pharmacy-driven post-exposure prophylaxis for HIV in Ohio”* which is being conducted at the University of Toledo under the direction of *Joan Duggan, MD.* The purpose of this study is to assess the knowledge, attitudes, and perspectives of medical students, medical residents, and attending physicians regarding HIV post-exposure prophylaxis (PEP) and the potential prescription of PEP by community pharmacists.

 **Description of Procedures:** This research study will be completed via an electronic survey which will be disseminated via an email from the Ohio State Medical Association (OSMA). If you choose to participate you will be asked to complete a single online survey which should take 10-15 minutes. You will be asked to complete various questions in which you will provide information on your knowledge and experience with PEP and your thoughts on ways PEP may be accessed by those in need. Additionally, there will be some questions that will provide a little more information about you (no personal information or identifiers will be collected).

 **Potential Risks:** Although minimal, a potential risk of participating in the research includes breach of
 confidentiality. To minimize this risk, the data is anonymous as no personal identifiers are being collected. Additionally, results for all subjects are combined and then analyzed.

 **Potential Benefits:** The only direct benefit to you if you participate in this research may be that you will learn about how *online surveys* are run and you may learn more about *the thoughts of your colleagues on PEP and its accessibility.* The field of HIV prevention may benefit from this research by gaining insight on the perspectives on PEP and its delivery by potential providers. Others may benefit by learning about the results of this research.

 **Confidentiality:** The data collected for this survey is anonymous as no personal information/identifers is
 being collected. The survey is not linked to you and efforts are made to prevent others from knowing of your participation. Additionally, results for all participants are combined and then analyzed.

 **Voluntary Participation:** The de-identified information collected may be used for future research
 purposes. As a reminder, your participation in this research is voluntary. Your refusal to participate in this
 study and doing so will not affect your relationship with The University of Toledo. You may skip any questions that you may be uncomfortable answering. You may discontinue participation at any time.

 **Contact Information:** If you have any questions at any time before, during or after your participation
 you should contact a member of the research team: Eric Sahloff, PharmD, 419-383-1962; Joan Duggan,
 MD, 419-383-4328.

 If you have questions beyond those answered by the research team or your rights as a research subject or research-related injuries, the Chairperson of the SBE Institutional Review Board may be contacted through the Human Research Protection Program on the main campus at (419) 530-6167.

 **CONSENT SECTION – Please read carefully**
 You are making a decision whether or not to participate in this research study. ***By clicking the blue arrow (ie, starting the survey)*** you indicate that you have read the information provided above, you have had all your questions answered, and you have decided to take part in this research. You may take as much time as necessary to think it over.

 By participating in this research, you confirm that you are at least 18 years old.

End of Block: Disclaimer

Start of Block: Demographics

**Q1** Age

________________________________________________________________

**Q2** Gender

o Male (1)

o Female (2)

o Non-binary / third gender (3)

o Prefer not to say (4)

o Other: (5) __________________________________________________

**Q24** Sexual orientation

o Straight, that is, not gay or lesbian (1)

o Lesbian or Gay (2)

o Bisexual (3)

o I use a different term [free-text] (4) __________________________________________________

**Q8** What is your race and ethnicity?

o Hispanic (1)

o White alone, non-Hispanic (2)

o Black or African American alone, non-Hispanic (3)

o American Indian and Alaska Native alone, non-Hispanic (4)

o Asian alone, non-Hispanic (5)

o Native Hawaiian and Other Pacific Islander alone, non-Hispanic (6)

o Some Other Race alone, non-Hispanic (7)

o Multiracial, non-Hispanic (8)

**Q3** What is your current status of practice?

o Retired / Non-direct patient care employment (1)

o Attending (6)

o Fellow (2)

o Resident (3)

o Medical Student (4)

**Q25** What is the zip code of your current medical practice or current medical training?
________________________________________________________________

End of Block: Demographics

Start of Block: Student Specific Questions

Display This Question:

If… What is your current status of practice? = Medical Student

**Q39** The following questions are about post-exposure prophylaxis (PEP) for HIV prevention. PEP involves providing an individual with 28 days of antiretroviral medication within 72 hours (ideally within 2 – 24 hours) after a potential HIV exposure in order to prevent HIV infection. Healthcare workers receive PEP after an occupational bloodborne pathogen exposure. Non-healthcare workers receive PEP after a sexual exposure or injection exposure. The type of medication and duration of therapy are the same in both settings. PEP is safe and effective in both settings when started in a timely manner and used as directed.

Display This Question:

If… What is your current status of practice? = Medical Student

**Q40** Were you aware of this medical information prior to reading the above paragraph?

o I was **not** aware of this information (1)

o I was aware of **some** of this information (2)

o I was aware of **most** of this information (3)

o I was aware of **all** of this information (4)

Display This Question:

If… What is your current status of practice? = Medical Student

**Q41** How do you rank your knowledge/understanding of PEP?

o I have no experience (1)

o Novice (2)

o Intermediate (3)

o Advanced (4)

o Expert (5)

Display This Question:

If… What is your current status of practice? = Medical Student

**Q9** Have you participated in the care of people living with HIV in the education setting?

o No, none (1)

o Yes, a few ( (2)

o Yes, many (>10 patients) (3)

Display This Question:

If… What is your current status of practice? = Medical Student

**Q10** Have you participated in the care of patients receiving post exposure prophylaxis for HIV in the educational setting?

o No, none (1)

o Yes, a few ( (2)

o Yes, many (>10 patients) (3)

End of Block: Student Specific Questions

Start of Block: Residents

Display This Question:

If… What is your current status of practice? = Resident

**Q12** What specialty are you currently training in?

▼ Allergy and Immunology (1) ... Urology (24)

Display This Question:

If… What is your current status of practice? = Resident

**Q47** The following questions are about post-exposure prophylaxis (PEP) for HIV prevention. PEP involves providing an individual with 28 days of antiretroviral medication within 72 hours (ideally within 2 – 24 hours) after a potential HIV exposure in order to prevent HIV infection. Healthcare workers receive PEP after an occupational bloodborne pathogen exposure. Non-healthcare workers receive PEP after a sexual exposure or injection exposure. The type of medication and duration of therapy are the same in both settings. PEP is safe and effective in both settings when started in a timely manner and used as directed.

Display This Question:

If… What is your current status of practice? = Resident

**Q46** Were you aware of this medical information prior to reading the above paragraph?

o I was **not** aware of this information (1)

o I was aware of **some** of this information (2)

o I was aware of **most** of this information (3)

o I was aware of **all** of this information (4)

Display This Question:

If What is your current status of practice? = Resident

**Q45** How do you rank your knowledge/understanding of PEP?

o I have no experience (1)

o Novice (2)

o Intermediate (3)

o Advanced (4)

o Expert (5)

Display This Question:

If… What is your current status of practice? = Resident

**Q13** Over the last year, how many patients have you cared for that are HIV positive?

o None (1)

o 1-10 (2)

o 11-25 (3)

o 26-50 (4)

o >50 (5)

Display This Question:

If… What is your current status of practice? = Resident

**Q34** In the past year, how many patients have you cared for that have been eligible for or prescribed PEP?

o None (1)

o 1-10 (2)

o 11-25 (3)

o 26-50 (4)

o >50 (5)

End of Block: Residents

Start of Block: Fellows/Attendings

Display This Question:

If… What is your current status of practice? = Fellow

Or What is your current status of practice? = Attending

Q17 What is your specialty?

▼ Allergy and Immunology (1) ... Urology (24)

Display This Question:

If… What is your current status of practice? = Fellow

Or What is your current status of practice? = Attending

And What is your specialty? = Anesthesiology

Q57 What is your sub - specialty?

▼ NA (1) ... Sleep Medicine (8)

Display This Question:

If What is your current status of practice? = Fellow

Or What is your current status of practice? = Attending

And What is your specialty? = …

Display This Question:

If… What is your current status of practice? = Attending

**Q20** In what setting do you primarily practice?

o Inpatient (1)

o Outpatient (2)

o Urgent Care (3)

o ER (4)

Display This Question:

If… What is your current status of practice? = Attending

Or What is your current status of practice? = Fellow

**Q44** The following questions are about post-exposure prophylaxis (PEP) for HIV prevention. PEP involves providing an individual with 28 days of antiretroviral medication within 72 hours (ideally within 2 – 24 hours) after a potential HIV exposure in order to prevent HIV infection. Healthcare workers receive PEP after an occupational bloodborne pathogen exposure. Non-healthcare workers receive PEP after a sexual exposure or injection exposure. The type of medication and duration of therapy are the same in both settings. PEP is safe and effective in both settings when started in a timely manner and used as directed.

Display This Question:

If What is your current status of practice? = Attending

Or What is your current status of practice? = Fellow

**Q43** Were you aware of this medical information prior to reading the above paragraph?

o I was **not** aware of this information (1)

o I was aware of **some** of this information (2)

o I was aware of **most** of this information (3)

o I was aware of **all** of this information (4)

Display This Question:

If What is your current status of practice? = Attending

Or What is your current status of practice? = Fellow

**Q42** How do you rank your knowledge/understanding of PEP?

o I have no experience (1)

o Novice (2)

o Intermediate (3)

o Advanced (4)

o Expert (5)

Display This Question:

If… What is your current status of practice? = Attending

Or What is your current status of practice? = Fellow

**Q32** Over the last year, how many patients have you cared for that are HIV positive?

o None (1)

o 1-10 (2)

o 11-25 (3)

o 26-50 (4)

o >50 (5)

Display This Question:

If… What is your current status of practice? = Fellow

Or What is your current status of practice? = Attending

**Q36** In the past year, how many patients have you cared for that have been eligible for or prescribed PEP?

o None (1)

o 1-10 (2)

o 11-25 (3)

o 26-50 (4)

o >50 (5)

End of Block: Fellows/Attendings

Start of Block: Retired

Display This Question:

If… What is your current status of practice? = Retired / Non-direct patient care employment

**Q31** What is your specialty?

▼ Allergy and Immunology (1) ... Urology (24)

Display This Question:

If… What is your current status of practice? = Retired / Non-direct patient care employment

**Q28** How many years have you been retired from the active practice of medicine?

o 0-5 years (1)

o 6-10 years (2)

o >10 years (3)

Display This Question:

If What is your current status of practice? = Retired / Non-direct patient care employment

**Q48** The following questions are about post-exposure prophylaxis (PEP) for HIV prevention. PEP involves providing an individual with 28 days of antiretroviral medication within 72 hours (ideally within 2 – 24 hours) after a potential HIV exposure in order to prevent HIV infection. Healthcare workers receive PEP after an occupational bloodborne pathogen exposure. Non-healthcare workers receive PEP after a sexual exposure or injection exposure. The type of medication and duration of therapy are the same in both settings. PEP is safe and effective in both settings when started in a timely manner and used as directed.

Display This Question:

If… What is your current status of practice? = Retired / Non-direct patient care employment

Q49 Were you aware of this medical information prior to reading the above paragraph?

o I was **not** aware of this information (1)

o I was aware of **some** of this information (2)

o I was aware of **most** of this information (3)

o I was aware of **all** of this information (4)

Display This Question:

If… What is your current status of practice? = Retired / Non-direct patient care employment

**Q50** How do you rank your knowledge/understanding of PEP?

o I have no experience (1)

o Novice (2)

o Intermediate (3)

o Advanced (4)

o Expert (5)

Display This Question:

If… What is your current status of practice? = Retired / Non-direct patient care employment

**Q35** During active practice, in an average year, how many patients did you care for that are HIV positive?

o None (1)

o 1-10 (2)

o 11-25 (3)

o 26-50 (4)

o >50 (5)

Display This Question:

If … What is your current status of practice? = Retired / Non-direct patient care employment

**Q14** During active practice, in an average year, how many patients did you care for that have been eligible for or prescribed PEP.

o None (1)

o 1-10 (2)

o 11-25 (3)

o 26-50 (4)

o >50 (5)

End of Block: Retired

Start of Block: PEP opinions

**Q78** For medications requiring urgent administration, federal or certain state regulations allow pharmacists to provide immunizations* and emergency medications** without a prescription under defined conditions. In Ohio, these include: Emergency naloxone, Immunizations ,Emergency contraception, Nirmaltrovir/ritonavir (Paxlovid® for Covid-19 under FDA guidance). The following questions relate to pharmacy prescribing in Ohio.
 * ORC 4729.283 https://codes.ohio.gov/ohio-revised-code/section-4729.283
 ** OCR Section 4729.41 https://codes.ohio.gov/ohio-revised-code/section-4729.41

**Q4** Please indicate your response to the following statements.

|  |  |  |  |  |  |
| --- | --- | --- | --- | --- | --- |
| How **important** is it to provide timely post-exposure prophylaxis to people exposed to HIV through sex or injection drug use (IE, non-occupational exposure)? (25) | o Definitely not important (1) | o Somewhat not important (2) | o Neutral (3) | o Somewhat important (4) | o Definitely important (5) |
| How **important** is it to provide post-exposure prophylaxis to people with occupational exposure to HIV? (26) | o Definitely not important (1) | o Somewhat not important (2) | o Neutral (3) | o Somewhat important (4) | o Definitely important (5) |
| Will allowing pharmacists to prescribe PEP **improve** patient access? (32) | o Definitely will not improve (1) | o Probably will not improve (2) | o Neutral (3) | o Probably will improve (4) | o Definitely will improve (5) |
| Is it **safe** for pharmacists to prescribe medications requiring urgent administration such as Narcan or Paxlovid to patients who have not yet been seen by a medical provider such as physician or physician extender? (28) | o Definitely no (1) | o Somewhat no (2) | o Neutral (3) | o Somewhat yes (4) | o Definitely yes (5) |
| Is it **appropriate** for pharmacists to prescribe medications requiring urgent administration such as Narcan or Paxlovid to patients who have not yet been seen by a medical provider such as physician or physician extender? (29) | o Definitely no (1) | o Somewhat no (2) | o Neutral (3) | o Somewhat yes (4) | o Definitely yes (5) |
| Is it **safe** for pharmacists to prescribe PEP to patients who have not yet been seen by a medical provider such as physician or physician extender? (30) | o Definitely no (1) | o Somewhat no (2) | o Neutral (3) | o Somewhat yes (4) | o Definitely yes (5) |
| Is it **appropriate** for pharmacists to prescribe PEP to patients who have not yet been seen by a medical provider such as physician or physician extender? (31) | o Definitely no (1) | o Somewhat no (2) | o Neutral (3) | o Somewhat yes (4) | o Definitely yes (5) |

**Q53** What do you think will be the impact on appropriate use of PEP if pharmacists are allowed to prescribe non-occupational PEP?

o Decrease inappropriate use (1)

o No impact on inappropriate use (2)

o Increase inappropriate use (3)

**Q52** What do you think will be the impact on the diagnosis of HIV and/or sexually transmitted infections (STIs) if pharmacists prescribe non-occupational PEP

o Decrease diagnosis of HIV and/or STIs (1)

o No impact on the diagnosis of HIV and/or STIs (2)

o Increase diagnosis of HIV and/or STIs (3)

**Q51** What do you think will be the impact on medical follow up for care for people at risk of HIV infection if pharmacists prescribe non-occupational PEP?

o Decrease follow up (1)

o No impact on follow up (2)

o Increase follow up (3)

**Q16** The following type of system for PEP would work well in Ohio:

o Pharmacists should be allowed to give a 28-day script for PEP using a protocol approved by the State Board of Pharmacy. (1)

o Pharmacist should be allowed to give a 7-day script for PEP using a protocol approved by the State Board of Pharmacy that has a mandatory requirement for referral/linkage to medical care. (2)

o Other (3)

o Pharmacists should not be allowed to prescribe PEP in Ohio. (4)

**Q87** Patients should be able to access PEP at which of the following locations?

|  | No (1) | Yes (2) |
| --- | --- | --- |
| Emergency Department (1) | o | o |
| Health Department (2) | o | o |
| Pharmacy (3) | o | o |
| Primary Care Provider (4) | o | o |
| Specialty Clinics (5) | o | o |
| Urgent Care (6) | o | o |
| Other (7) | o | o |

Q17 Do you have any other comments on pharmacist prescribing PEP in Ohio? (300 character limit)

________________________________________________________________

________________________________________________________________

________________________________________________________________

End of Block: PEP opinions
